# Regulatory Master Genes Identification and Drug Repositioning by Integrative mRNA-miRNA Network Analysis for Acute Type A Aortic Dissection

**DOI:** 10.3389/fphar.2020.575765

**Published:** 2021-01-21

**Authors:** Junjun Fang, Zongfu Pan, Hao Yu, Si Yang, Xiaoping Hu, Xiaoyang Lu, Lu Li

**Affiliations:** ^1^ Department of Clinical Pharmacy, The First Affiliated Hospital, College of Medicine, Zhejiang University, Zhejiang, China; ^2^ Surgical Intensive Critical Care Unit, The First Affiliated Hospital, College of Medicine, Zhejiang University, Zhejiang, China; ^3^ Department of Pharmacy, Zhejiang Provincial People’s Hospital, Zhejiang, China; ^4^ Thoracic Surgery, Sir Run Run Shaw Hospital, College of Medicine, Zhejiang University, Zhejiang, China

**Keywords:** acute type a aortic dissection, differentially expressed genes, weighted correlation network analysis, drug repositioning, differentially expressed miRNA

## Abstract

Acute type A aortic dissection (ATAAD) is a life-threatening disease. The understanding of its pathogenesis and treatment approaches remains unclear. In the present work, differentially expressed genes (DEGs) from two ATAAD datasets GSE52093 and GSE98770 were filtered. Transcription factor TEAD4 was predicted as a key modulator in protein-protein interaction (PPI) network. Weighted correlation network analysis (WGCNA) identified five modules in GSE52093 and four modules in GSE98770 were highly correlated with ATAAD. 71 consensus DEGs of highly correlated modules were defined and functionally annotated. L1000CDS^2^ was executed to predict drug for drug repositioning in ATAAD treatment. Eight compounds were filtered as potential drugs. Integrative analysis revealed the interaction network of five differentially expressed miRNA and 16 targeted DEGs. Finally, master DEGs were validated in human ATAAD samples and AD cell model *in vitro*. TIMP3 and SORBS1 were downregulated in ATAAD samples and AD cell model, while PRUNE2 only decreased *in vitro*. Calcium channel blocker and glucocorticoid receptor agonist might be potential drugs for ATAAD. The present study offers potential targets and underlying molecular mechanisms ATAAD pathogenesis, prevention and drug discovery.

## Introduction

Aortic dissection (AD) is a potentially critical break in the lining of the main arterial outflow from the heart. It comprises 85%–95% of all acute aortic syndromes (Bossone et al., 2018). The morbidity of AD is increasing in the last 16 years (Olsson et al., 2006), and current studies in different countries have found that the incidence of AD occurs in 3–6 cases per 100,000 people per year (Nienaber and Clough, 2015; Mussa et al., 2016). AD can be classified using Stanford or DeBakey systems. The Stanford system divides dissections according to whether the ascending aorta is involved (type A) or not (type B) (Golledge and Eagle, 2008). In the International Registry of Acute Aortic Dissection series, acute type A aortic dissection (ATAAD) is more common than type B (Evangelista et al., 2018). ATAAD is more prone to have the complication of acute heart failure on presentation than type B (Mussa et al., 2016), and the in-hospital mortality rate of patients with type A is 22%, while type B is only 13% (Evangelista et al., 2018).

Surgery is widely regarded as the most important treatment for ATAAD. Surgical management was increased from 79% to 90% to treat ATAAD for the past 17 years (*p* < 0.001) (Pape et al., 2015), but the mortality of patients with ATAAD managed surgically was high (Hagan et al., 2000). Moreover, the medical mortality rate of those with ATAAD also remained high and did not change overtime (Evangelista et al., 2018). Drug treatment before the onset and after surgery would be beneficial to decrease mortality and recurrence of ATAAD. Drugs used for AD mainly aimed at controlling heart rate, lowering blood pressure and stabilizing plaque (Erbel et al., 2014). However, there is no public evidence proved to prevent the occurrence of AD (Bossone et al., 2018; Evangelista et al., 2018). Therefore, finding potential targets or compounds would be useful and promising approaches to ATAAD prevention or treatment.

Microarray technology has been widely used to diagnose disease, reveal the pathogenesis and discover new drug targets in recent years. Several studies reported new targets and underlying mechanisms in ATAAD using microarray technology (Weis-Muller et al., 2006; Cheuk and Cheng, 2011; Pan et al., 2014; Kimura et al., 2017; Pan et al., 2017; Wang L et al., 2017). However, the small amounts of samples and sample heterogeneity limited the understanding of ATAAD in these studies. Therefore, integrating microarray data can provide reliable and valuable signals for ATAAD. Drug repositioning refers to the development of existing drugs or compounds for new indications (Ashburn and Thor, 2004). The gene-phenotype connection has been used to predict novel disease signatures and potential targets for drugs, and large-scale data derived from systems biology were integrated by bioinformatics and cheminformatics tools for drug repositioning strategies (Karaman and Sippl, 2018). The library of integrated network-based cellular signatures (LINCS) L1000 dataset consists of over a million gene expression profiles of chemically perturbed human cell lines. Characteristic direction signature search engine L1000CDS^2^ based on L1000 dataset provides prioritization of thousands of small-molecule signatures, and could be used in drug repositioning for therapeutics (Duan et al., 2016). MiRNAs inhibit target mRNAs at post transcriptional level, and take important roles in the AD pathological process (Sbarouni and Georgiadou, 2018a). Analysis of miRNA-mRNA network of ATAAD could provide more reliable potential genes in ATAAD pathogenesis, prevention and drug discovery.

In the present work, two datasets GSE52093 (Pan et al., 2014) and GSE98770 (Kimura et al., 2017) were downloaded from the public database National Center for Biotechnology Information (NCBI) Gene Expression Omnibus (GEO) (https://www.ncbi.nlm.nih.gov/geo/) for integrative analysis. Differentially expressed genes (DEGs) from ascending aorta samples of both datasets were filtered. Protein-protein interaction (PPI) network and transcription factor network identification were applied in these DEGs. Weighted correlation network analysis (WGCNA) constructed the coexpression network and identified the highly correlated modules with ATAAD in two datasets respectively. DEGs in highly correlated modules were defined as consensus DEGs, and selected for Gene Ontology (GO) and Kyoto Encyclopedia of Genes and Genomes (KEGG) pathway enrichment analyses. Furthermore, L1000CDS^2^ was applied to predict drugs for ATAAD treatment using consensus DEGs. Finally, differentially expressed miRNA as well as their predicted target genes were analyzed. Targeted DEGs were identified with predicted targets and consensus DEGs. As a result, our study aims to highlight the insight into pathological mechanism, prevention and treatment of ATAAD.

## Materials and Methods

### Microarray Data Information

The gene expression datasets GSE52093 and GSE98770 were obtained from NCBI GEO. All samples of both datasets were obtained from ascending aorta tissues. GSE52093 performing with mRNA microarrays contained five normal organ donors and seven ATAAD patients (Pan et al., 2014). GSE98770 contained 11 dissected ascending aorta samples of five normal organ donors and six ATAAD patients (Kimura et al., 2017). Gene expression profiling of GSE98770 was performed with mRNA and miRNA microarrays.

### DEGs Identification

Raw data of GSE52093 and GSE98770 were obtained from GEO. These two datasets are based on different platforms, therefore the methods to identify the DEGs were different. The dataset of GSE52093 was based on the platform GPL10558 (Illumina HumanHT-12 V4.0 expression beadchip), and the DEGs were analyzed based on the limma package in R. Limma package identified the DEGs using Empirical Bayes test. The dataset of GSE98770 was based on the platform GPL14550 (Agilent-028004 SurePrint G3 Human GE 8 × 60K Microarray) for mRNA microarray, and were analyzed for DEGs identification using unpaired *t* test with Benjamini-Hochberg false discovery rate correction by Agilent GeneSpring GX (version 11.5, Genomax Technologies Pte Ltd., Singapore). Statistically significant DEGs of both datasets were defined with an FDR <0.05 and |fold change (FC)|>1.5 as the cut-off criteria. The common DEGs in two datasets were identified by Venn diagram.

### PPI Network and Master Regulator Analysis

A PPI network of the DEGs was analyzed by STRING database (http://string-db.org), and a confidence score > 0.9 (highest confidence) was set as significant. The FCs of common DEGs were presented as averages of GSE52093 and GSE98770. PPI network was then visualized by Cytoscape software (version 3.7.2, The Cytoscape Consortium, New York, NY, United States).

Cytoscape plugin iRegulon was used to identify TFs of the PPI network. The iRegulon was set as default. TFs with NES ≥3 and targeted more than 50% of the network nodes were identified as master TFs and then selected to construct the regulatory network. The expression levels of master TFs and all nodes in the PPI network were calculated and applied to Pearson correlation analysis.

### WGCNA to Construct Coexpression Networks

The weighted correlations between ATAAD and all genes in GSE52093 or GSE98770 were analyzed by WGCNA package respectively as previously described (Li et al., 2018). The soft threshold power was calculated automatically. Modules are distinguished by colors. The correlation relationships between modules and ATAAD were then calculated to elucidate the highly correlated modules with ATAAD. The correlation coefficients and *p*-values were represented in the module-trait relationships. Modules with |correlation coefficient| > 0.5 were set as highly correlated modules with ATAAD.

### Consensus DEGs Filtration and Functional Pathway Enrichment Analysis

Consensus DEGs were filtered by overlapping the DEGs and genes in highly correlated modules with ATAAD in two datasets using Venn diagram. KEGG pathway enrichment and GO biological process enrichment were analyzed by Cytoscape using ClueGO and CluePedia. ClueGO was set as follows: kappa score threshold to 0.4, the *p*-value to 0.05, two-sided hypergeometric test, Bonferroni step down.

### Drug Repositioning Identification

L1000CDS^2^ (http://amp.pharm.mssm.edu/l1000cds2/#/index) is an advanced version of Connectivity Map with significantly increased drug treatments, cell types and gene signatures based on LINCS L1000 high-throughput technology. Consensus DEGs were inputted for drug repositioning. Compounds with overlap >0.1 were screened as potential drugs. MOA for potential drugs was provided by L1000 Fireworks Display (http://amp.pharm.mssm.edu/l1000fwd).

### Differentially Expressed miRNA Identification

The dataset of GSE98770 was based on platform GPL17660 (Agilent-031181 Unrestricted Human miRNA V16.0 Microarray 030840) for miRNA microarray. The raw data were analyzed by NCBI web tool GEO2R (https://www.ncbi.nlm.nih.gov/geo/geo2r/) to identify differentially expressed miRNA. GEO2R performs comparisons using the limma R packages from the Bioconductor project. Significance was defined with FDR < 0.05 and |log_2_ FC| > 1 as the cut-off criteria.

### miRNA-Targeted DEG Interaction Network Analysis

The potential gene targets of differentially expressed miRNA were predicted by miRWalk Target Mining page (http://mirwalk.umm.uni-heidelberg.de/search_mirnas/). The Target Mining page provides an advanced search option for several miRNAs. The score was set as ≥0.95. Potential targets which also act as consensus DEGs were filtered and identified as targeted DEGs. miRNA-targeted DEG interaction network was visualized by Cytoscape software.

### Human Ascending Aorta Tissue Samples Obtain and Establishment of AD Models *In Vitro*

Human ascending aorta tissue samples obtained from six ATAAD patients and four non-ATAAD patients who underwent surgical resection. Non-ATAAD samples were normal tissues surrounding the cut samples from surgical donors. All specimens from ATAAD patients had a pathological diagnosis at the time of assessment. Studies were conducted under the prior informed consent procedure and with written consent from the human ethics committee of the first affiliated hospital (No. 2019-1493), college of medicine, Zhejiang University, China.

Human VSMCs were treated with 0.1 μm AngII for 12 h to mimic AD models *in vitro* (Li et al., 2017). Cells then were collected for DEGs validation.

### Validation of Master DEGs with Quantitative Reverse Transcription Polymerase Chain Reaction

The overlapped genes of consensus DEGs regulated by predicted drugs and targeted DEGs of differentially expressed miRNAs were identified as master DEGs. Master DEGs were validated by quantitative reverse transcription polymerase chain reaction (qRT-PCR) as previously described (Li et al., 2018). Total RNA was extracted by RNAsimple Total RNA Kit (Tian Gen, Beijing, China). cDNAs were conducted by PrimeScript TM RT reagent Kit (Takara Bio, Shiga, Japan). PCR amplifications were applied by TB Green TM Premix Ex Taq TM II (TaKaRa Bio). Measurement were normalized to GAPDH. All primer pairs are listed in Supplementary Table S1.

### Statistical Analysis

At least three independent cell experiments were carried out as repeats. Statistical analysis was performed by GraphPad Prism 6 (GraphPad Software Inc., San Diego, CA, United States). For human samples, statistical analysis was performed by unpaired *t* test. For cell samples, statistical analysis was performed by paired *t* test. A value of *p* < 0.05 was considered to be significant.

## Results

### Identification of DEGs in ATAAD

**Figure 1:**
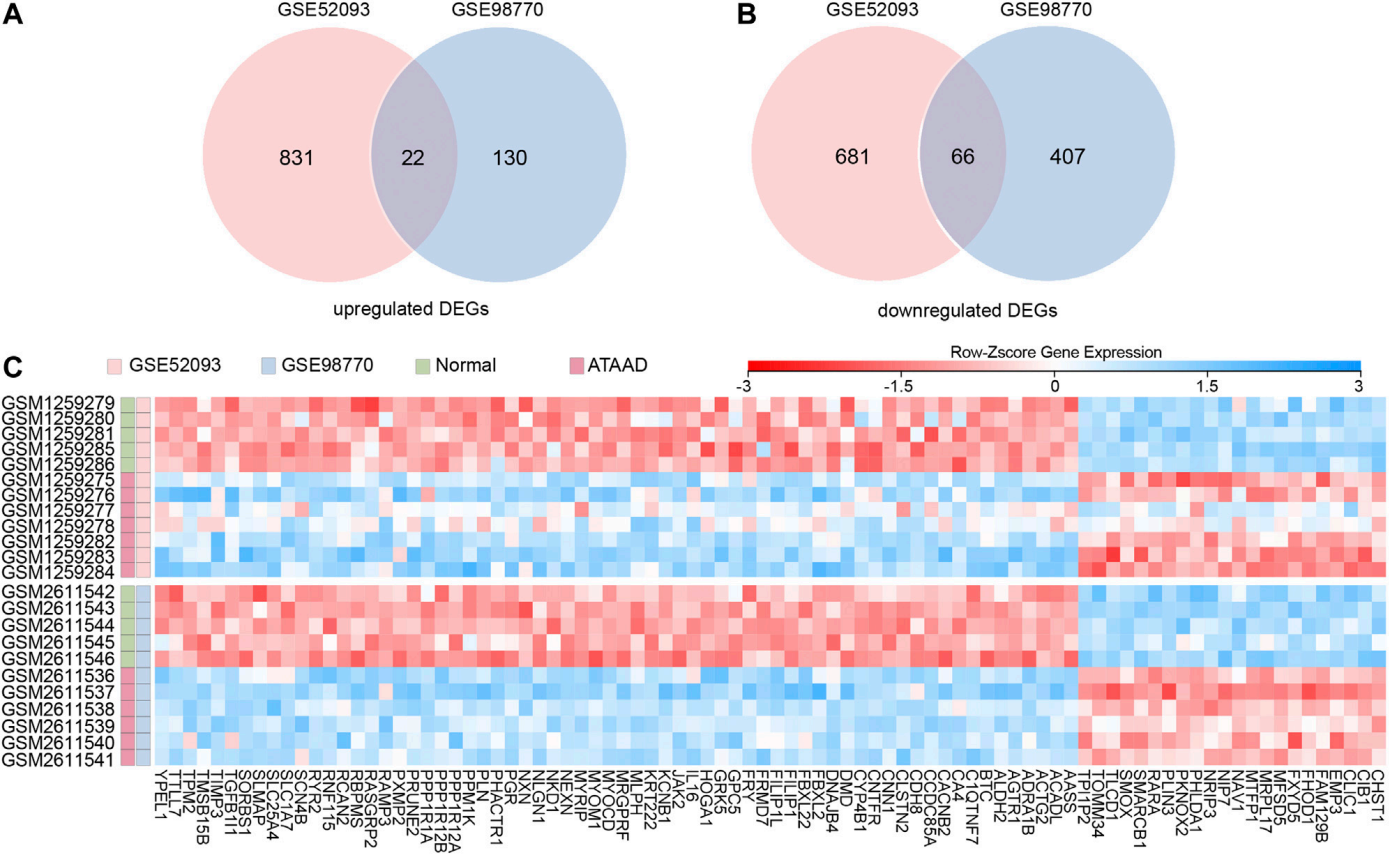
Identification of commonly changed DEGs in GSE52093 and GSE98770. **(A)**, **(B)** Venn diagram displayed the upregulated DEGs **(A)** and downregulated DEGs **(B)** in both two datasets. **(C)** Heatmap of the 88 common DEGs in two datasets. Red: up-regulation; blue: down-regulation. Gene probe values were normalized by row Z-score.

GSE52093 and GSE98770 were obtained from NCBI GEO. All of the samples from these datasets were obtained from ascending aorta. DEGs were defined as false discovery rate (FDR) < 0.05, and |fold change (FC)| > 1.5. A total of 1600 DEGs were screened in GSE52093, while 625 DEGs were filtered from GSE98770. As a result, 22 upregulated DEGs (Figure 1A) and 66 downregulated DEGs (Figure 1B) were found in both datasets. The expressions of these 88 common DEGs in both two datasets were displayed in a heatmap (Figure 1C). As shown in Figure 1C, these DEGs could clearly distinguish ATAAD from normal samples.

### PPI Network and Transcription Factors (TFs) Analysis of DEGs in ATAAD

**Figure 2:**
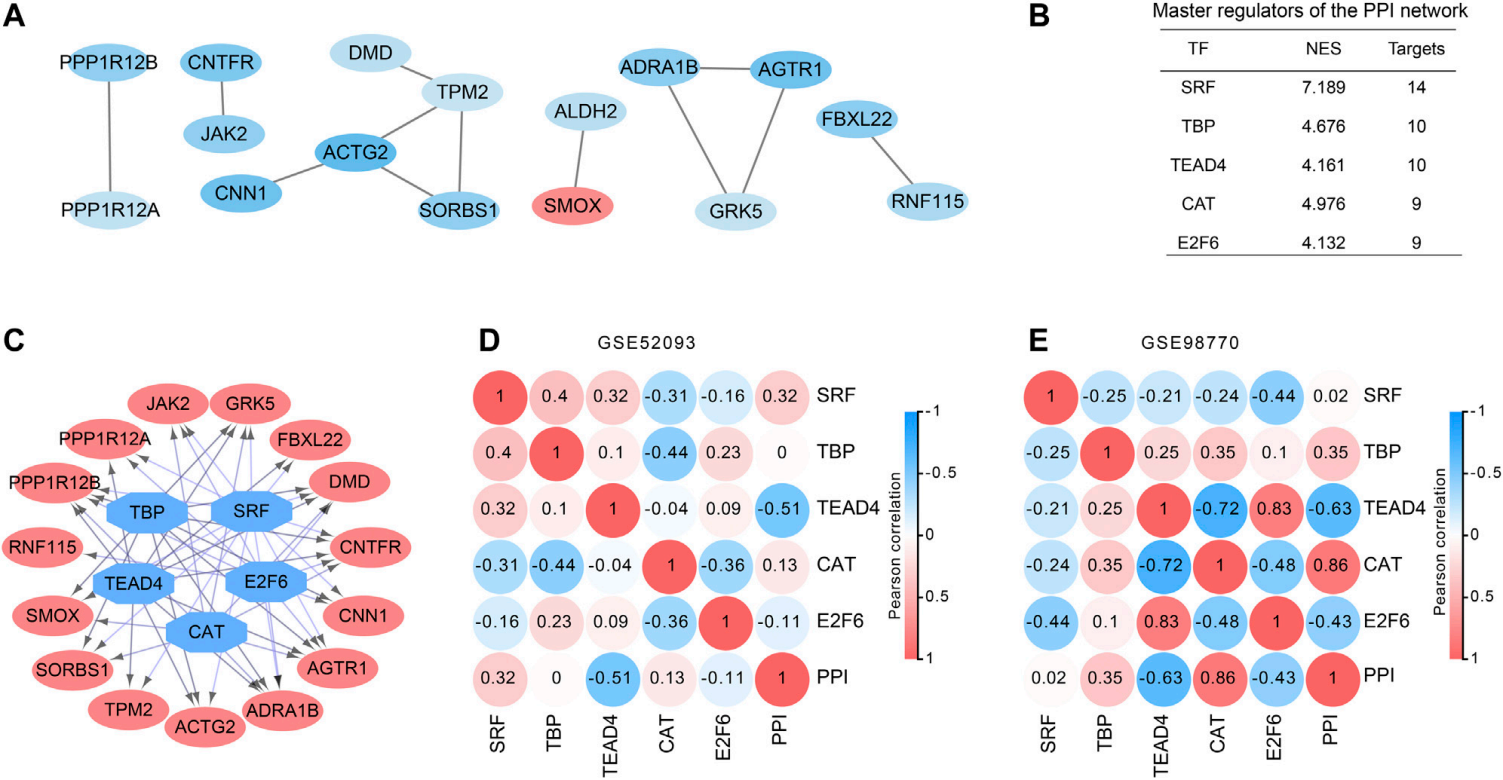
PPI and transcription factor network construction in DEGs of ATAAD. **(A)**. PPI network of commonly changed DEGs was constructed. Red indicated the upregulated DEGs and blue indicated the downregulated DEGs. **(B)** Master regulators of the PPI network. TFs with NES ≥3 and covered more than 50% of the network nodes were identified as master regulators. **(C)** Master regulator targeted network of the PPI network. Blue octagon nodes represent the predicted transcription factors. Red oval nodes represent transcription factor regulated genes. **(D)**, **(E)** Correlations between master regulators and PPI network. Pearson correlation coefficient was shown in the circles.

All 88 DEGs were analyzed by the STRING database to verify the functional connectivity. As a consequence, there were 16 nodes and 12 edges in the PPI network with the highest confidence score, which represented proteins and functional interactions (Figure 2A). ACTG2 (degree = 3) and TPM2 (degree = 3) had the highest degree scores in the PPI network. TFs in the network were then predicted by iRegulon plugin of cytoscape. Five TFs SRF, TBP, TEAD4, CAT and E2F6 which had a NES ≥ 3 and targeted more than 50% of the network nodes were identified as master TFs of PPI network and displayed in Figure 2B. Interaction network of the master TFs and potential targeted DEGs was constructed (Figure 2C). The correlations between master TFs and PPI network were shown in Figure 2D (GSE98770) and 2E (GSE52093). We found that in the expression profiles of GSE98770, TEAD4 (Pearson *r* = −0.63, *p* = 0.036) was negatively correlated with PPI network, and CAT (Pearson *r* = 0.86, *p* = 0.0008) was positively correlated with the PPI network at significant levels (Figure 2D). In the expression profiles of GSE52093, TEAD4 (Pearson *r* = −0.51, *p* = 0.087) was also negatively correlated with PPI network, even though the difference did not reach statistical significance. Therefore, we concluded that *TEAD4* might be a key TF in regulating PPI network of ATAAD.

### WGCNA Identified Highly Correlated Modules in ATAAD

**Figure 3:**
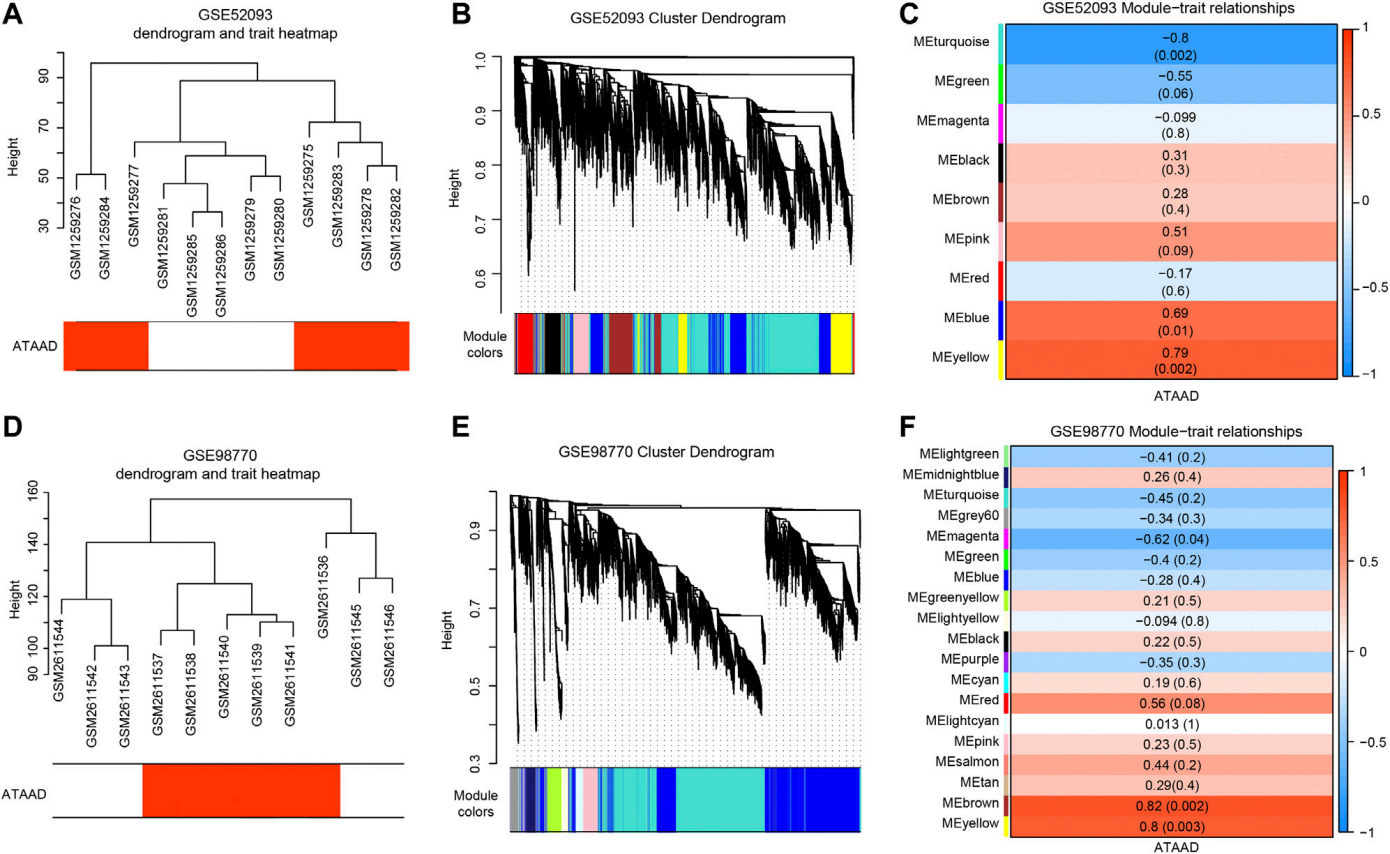
WGCNA detected the correlated modules with ATAAD in GSE52093 and GSE98770. **(A)**, **(D)** Sample dendrogram and trait heatmap of GSE52093 **(A)** and GSE98770 **(D)**. The red color represents ATAAD sample, while the white represents normal samples. **(B)**, **(E)** Construction of coexpression modules differentiated by colors. Each color indicated a correlated module of highly connected genes in GSE52093 **(B)** and GSE98770 **(E)**. The gray module contained unassigned genes. **(C)**, **(F)** Module and ATAAD relationships of GSE52093 **(C)** and GSE98770 **(F)** were displayed. Each row corresponds to a module eigengene. Each cell contains the corresponding correlation and the *p*-value in the parenthesis. Red represents a positive correlation and blue represents a negative correlation. Modules with |correlation coefficient| > 0.5 were set as highly correlated modules with ATAAD.

Weighted coexpression networks were analyzed by WGCNA in two datasets respectively. The sample dendrogram and ATAAD heatmap of GSE52093 and GSE98770 were shown in Figures 3A and 3D. *β* = 12 was selected for GSE52093, and *β* = 8 was selected for GSE98770. Altogether nine modules were identified by the hierarchical clustering dendrogram in GSE52093 (Figure 3B). Among these gene modules, turquoize module and green module were negatively correlated with ATAAD, while pink module, blue module and yellow module were positively correlated with ATAAD (Figure 3C). In GSE98770, a total of 19 distinct modules were identified (Figure 3E). Magenta module was found to be negatively related to ATAAD, while red module, brown module and yellow module were positively related to ATAAD (Figure 3F).

### Functional Pathway Enrichment in Consensus DEGs of ATAAD

**Figure 4:**
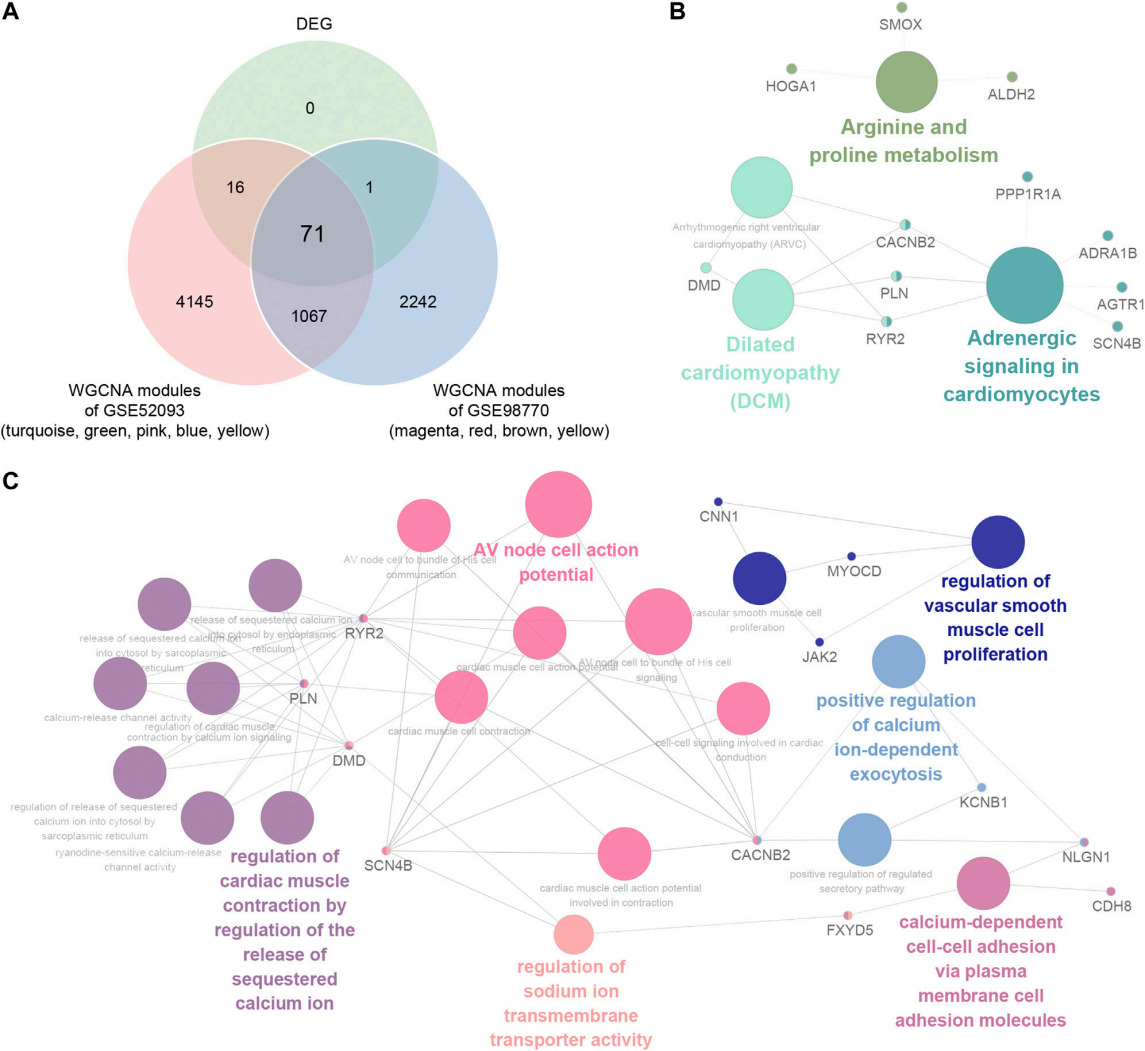
Identification and functional pathways annotation of consensus DEGs in ATAAD based on WGCNA. **(A)** Venn diagram showed the consensus DEGs included in highly correlated modules in GSE52093 and GSE98770. **(B)** KEGG pathway analysis of consensus DEGs. **(C)** Gene annotation enrichment analysis of GO biological process of consensus DEGs.

In order to elucidate the deep relationships between genes and ATAAD based on coexpression networks, we overlapped the DEGs and genes in highly correlated modules, and identified these genes as consensus DEGs. WGCNA indicated that turquoize, green, pink, blue and yellow modules of GSE52093 are highly correlated with ATAAD, while magenta, red, brown and yellow modules of GSE98770 are highly correlated with ATAAD (Figures 3E and 3F). 5,299 genes were included in highly correlated modules of GSE52093, and 3,381 genes were included in highly correlated modules of GSE98770. As a result, 71 consensus DEGs were filtered by Venn diagram (Figure 4A). The KEGG pathway analysis demonstrated that arginine and proline metabolism, dilated cardiomyopathy (DCM), and adrenergic signaling in cardiomyocytes were enriched in consensus DEGs (Figure 4B). GO biological process analysis showed that positive regulation of calcium ion-dependent exocytosis, regulation of cardiac muscle contraction by regulation of the release of sequestered calcium ion, calcium-dependent cell-cell adhesion via plasma membrane cell adhesion molecules, regulation of vascular smooth muscle proliferation, AV node cell action potential, and regulation of sodium ion transmembrane transporter activity were enriched in consensus DEGs (Figure 4C).

### Drug Repositioning for ATAAD

**Figure 5:**
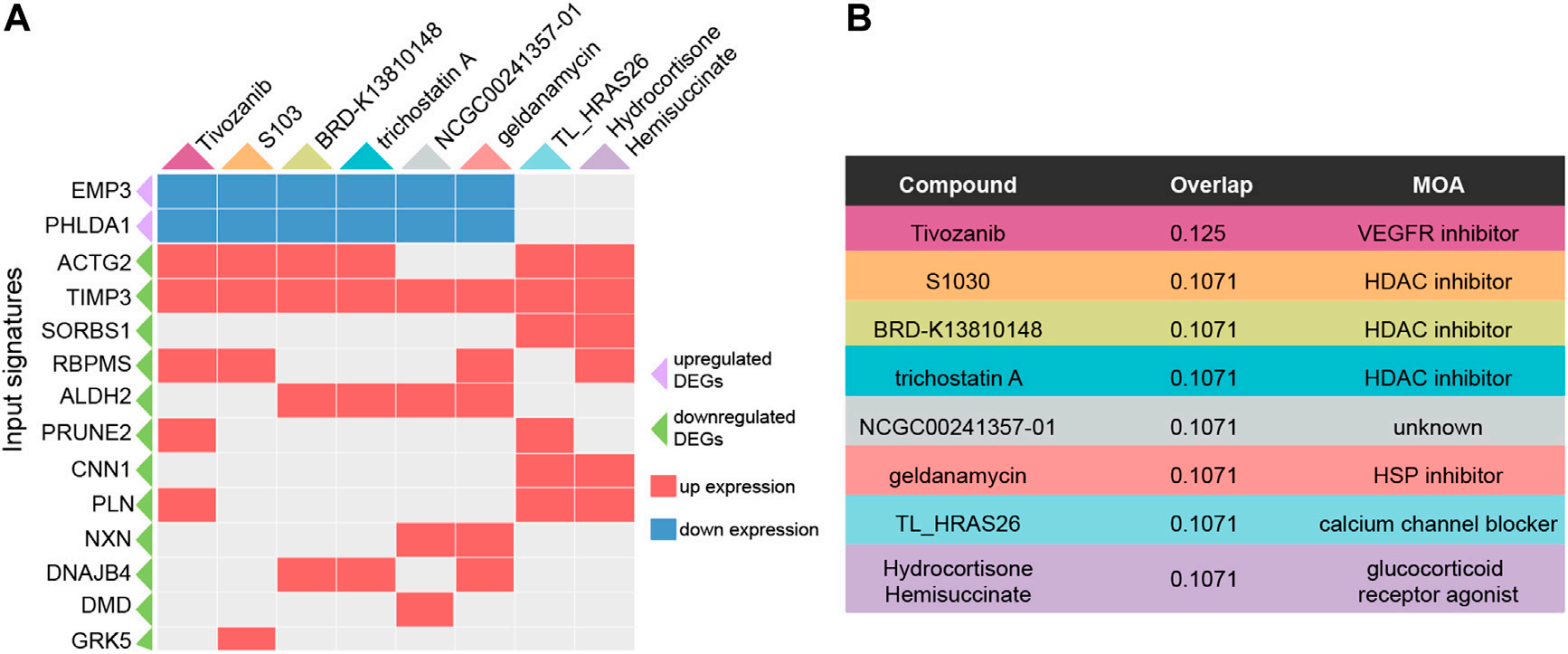
Predicted potential drugs for ATAAD through consensus DEGs by L1000CDS2. **(A)** Heatmap of predicted compounds and their modulated DEGs. Purple triangle indicated upregulated consensus DEGs, green triangle indicated downregulated consensus DEGs, red square indicated up expression after compounds stimulation, blue square indicated down expression after compounds stimulation. **(B)** MOA of the predicated compounds was provided by L1000FWD.

To explore potential drugs for ATAAD prevention or treatment after surgery, the characteristic direction signature search engine L1000CDS^2^ was applied. Consensus DEGs were inputted for drug repositioning. Depending on the identified 71 consensus DEGs, compounds or drugs that potentially reversed the gene signatures were acquired. Altogether 10 compounds with overlap > 0.1 were filtered as potential drugs. The heatmap of potential drugs and their modulated signatures were shown in Figure 5A. Upregulated consensus DEGs were downregulated by these compounds, while downregulated consensus DEGs were upregulated after compounds stimulation. Tivozanib regulated seven signatures among inputted consensus DEGs while others regulated six signatures. Among these compounds, tivozanib is a VEGFR inhibitor; S1030, BRD-K13810148, trichostatin A are HDAC inhibitors; geldanamycin is a HSP inhibitor; TL_HRAS26 is a calcium channel blocker; hydrocortisone hemisuccinate is a glucocorticoid receptor agonist (Figure 5B).

### miRNA and Targeted DEGs Interaction Network Regulation

**Figure 6:**
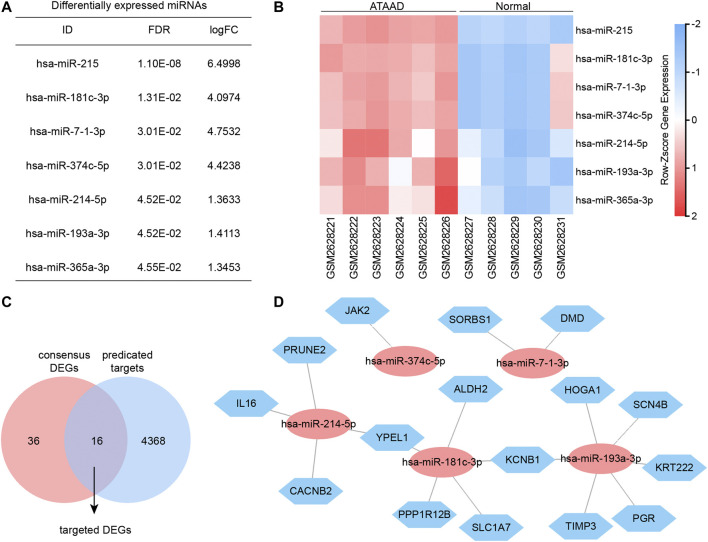
miRNA and mRNA interaction network. **(A)** Differentially expressed miRNAs of GSE98770 identified by GEO2R. **(B)** Heatmap of differentially expressed miRNA expression profiles. **(C)** Targeted DEGs were the overlapped genes obtained between target genes and consensus DEGs. **(D)** Interaction network of miRNA and targeted DEGs analyzed by miRWalk. Blue hexagons nodes indicated the potential targets which also act as consensus DEGs. Red ovals indicated the miRNA regulating these targeted DEGs.

Gene expression profiling of GSE98770 was also performed with miRNA microarrays. Here we identified the differentially expressed miRNAs by GEO2R. FDR < 0.05 was set as the cut-off criteria, and seven differentially expressed miRNAs were filtered (Figure 6A). The miRNA expression profiles were shown in the heatmap, and all of the miRNAs were upregulated in ATAAD (Figure 6B). We next predicted the potential target genes of these miRNA by miRWalk database. As a result, 4,384 potential targets were found. miRNA always regulated genes negatively. Since the miRNAs were all upregulated, therefore we predicted the targeted DEGs between target genes and 52 downregulated consensus DEGs. As a result, 16 target genes were also found in consensus DEGs (Figure 6C). Furthermore, interaction network of the miRNA and potential targeted DEGs was constructed (Figure 6D). These targeted DEGs were regulated by five of all differentially expressed miRNAs. JAK2 was regulated by miR-374c-5p. DMD and SORBS1 were regulated by miR-7-1-3p. KRT222, SCN4B, PGR, TIMP3, and HOGA1 were only regulated by miR-193a-3p. PRUNE2, CACNB2 and IL16 were only regulated by miR-214-5p. SLC1A7, ALDH2 and PPP1R12B were only regulated by miR-181c-3p. KCNB1 was regulated by miR-193a-3p and miR-181c-3p. YPEL1 was regulated by miR181c-3P and miR-214-5p.

### Validation of Master DEGs Associated With ATAAD

**Figure 7:**
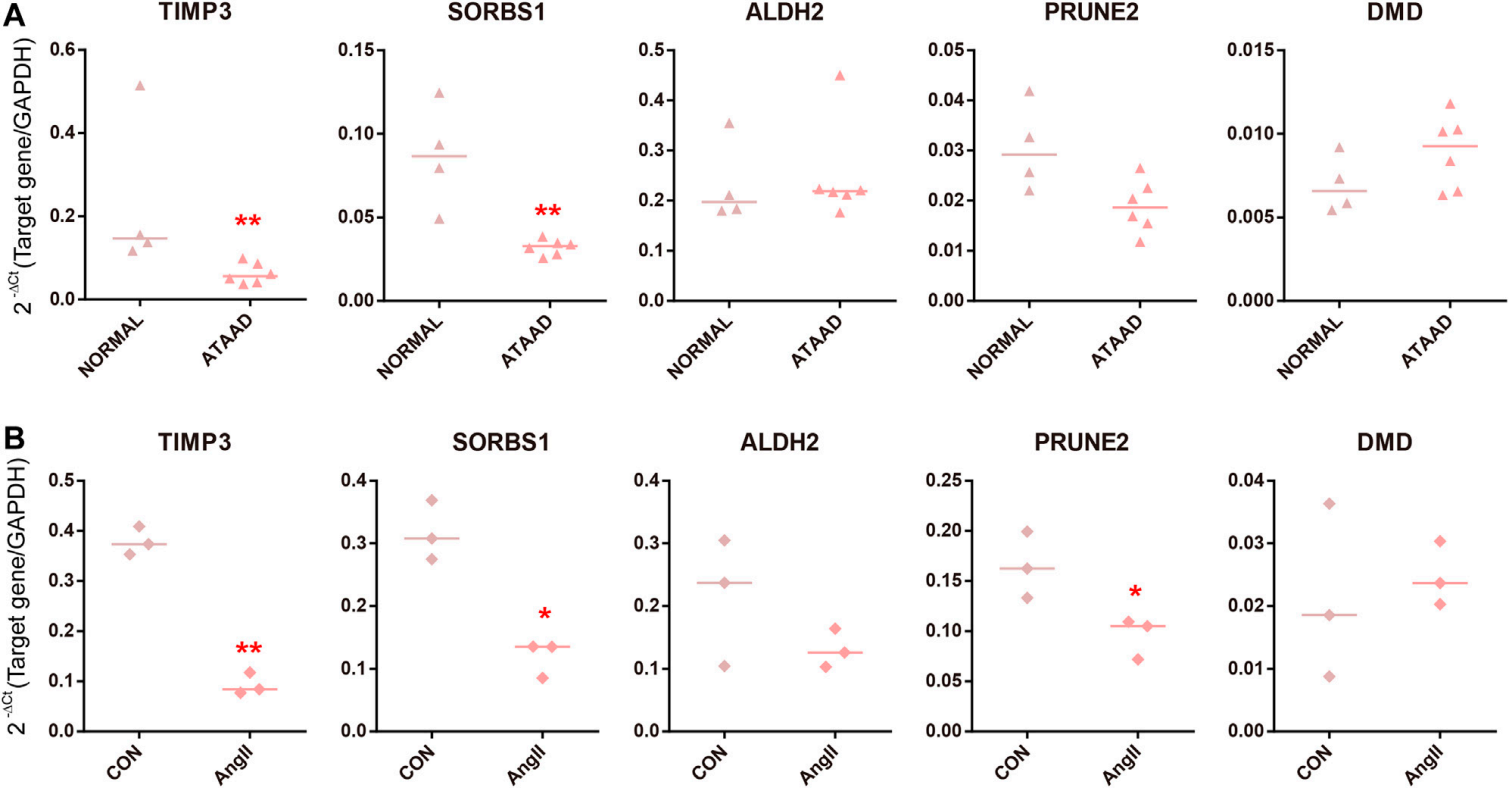
Validation of master DEGs in human ATAAD samples *in vivo* and AD cell model *in vitro*. **(A)** The mRNA levels in ascending aorta tissues in normal samples (*n* = 4) and ATAAD samples (*n* = 6). The line represented median. Statistical significance was set as *p < 0.05, **p < 0.01 vs. normal. **(B)** Human VSMCs were treated with AngII. The line represented median. *n* = 3. Statistical significance was set as *p < 0.05, **p < 0.01 vs. control.

Altogether 5 master DEGs, *TIMP3*, *PRUNE2*, *ALDH2*, *SORBS1* and *DMD* were included in consensus DEGs regulated by predicted drugs and targeted DEGs of differentially expressed miRNAs (Figures 5A and 6D). As a result, we found that *TIMP3* and *SORBS1* were decreased in ATAAD ascending aorta tissue samples (Figure 7A). Moreover, we established an AD cell model using human Vascular smooth muscle cells (VSMCs) under the stimulation of Angiotensin II (AngII). We demonstrated that *TIMP3, PRUNE2* and *SORBS1* were all downregulated in AD cell model (Figure 7B).

## Discussion

ATAAD is a common type of AD with high mortality (Evangelista et al., 2018). The limited understanding in pathogenesis and treatment approaches impaired ATAAD patients’ long term outcomes. Surgery is the most important treatment for ATAAD. Due to the high mortality, drug treatment before the onset and after surgery should be significant for reducing mortality and recurrence of ATAAD. Limited drugs used for AD mainly included beta-blockers, hypotensive drugs and statins (Erbel et al., 2014). Therefore, elucidating potential drug targets as well as the underlying mechanisms in ATAAD would contribute to highlight new strategies for ATAAD treatment. In the present study, we obtained two datasets GSE52093 and GSE98770 from public NCBI GEO. GSE52093 contained five normal organ donors and seven ATAAD patients (Pan et al., 2014), and GSE98770 contained five normal organ donors and six ATAAD patients (Kimura et al., 2017). All samples of the two datasets were acquired from dissected ascending aorta samples. Here we combined the two datasets in order to achieve more reliable and effective evidences for the pathogenesis and treatment of ATAAD by enlarging sample numbers.

In the present work, 88 DEGs were obtained totally. The PPI network identified *ACTG2* and *TPM2* had the highest degree scores in the PPI network. ACTG2 encodes actin gamma two; a smooth muscle actin. *TPM2* is a member of the actin filament binding protein family, and mainly expressed in slow, type 1 muscle fibers. A recent study found that the mRNA and protein expression levels of TPM2 were significantly upregulated in aorta of ATAAD patients (Zhong et al., 2020). However, our analysis indicated a decrease of *TPM2* in ATAAD samples. It remains further exploration to elucidate the effects of TPM2 in ATAAD. Until now, little is known about the roles of *ACTG2* in AD. Generally, Actins are highly conserved proteins, our result might indicate the cell motility alteration in cytoskeleton of ATAAD aorta smooth muscle cells.

Five TFs *SRF*, *TBP*, *TEAD4*, *CAT* and *E2F6* were predicted to regulate the PPI network of DEGs (Figure 2B). SRF, the serum response factor which had the highest NES among 5 TFs contributed to maintain the contractile phenotype of VSMCs (Xu et al., 2019) and smooth muscle differentiation (Horita et al., 2016). VSMC-specific EP4 deletion exacerbated angiotensin II-induced AD accompanied with decreased SRF transcriptional activity (Xu et al., 2019). Myocardin activates SRF, a conditional mutant of myocardin resulted in AD generation (Huang et al., 2015). Therefore, SRF might be a key TF in ATAAD pathogenesis. The relationships between other TFs and ATAAD have not been elucidated. *CAT* was positively correlated with the PPI network of GSE98770. CAT encodes catalase, a key antioxidant enzyme in the body’s defense against oxidative stress. Our result was consistent with the previous result that aortic ischemia-reperfusion increased the level of CAT (Kiris et al., 2008). *TEAD4* was negatively correlated with PPI network of both datasets (Figures 2D and 2E). *TEAD4* is a member of the transcriptional enhancer factor family of transcription factors. It participated in the 9p21.3 locus conferred risk of coronary artery disease in human aortic smooth muscle cells (Almontashiri et al., 2015). We conjectured that *TEAD4* might be a novel target of ATAAD.

The coexpression network analysis identified five highly correlated modules in GSE52093 and four highly correlated modules in GSE98770 with ATAAD respectively (Figure 3). 71 consensus DEGs were filtered from genes in highly correlated modules and DEGs (Figure 4A). Functional pathway enriched in adrenergic signaling in cardiomyocytes for KEGG pathway analysis (Figure 4B), and positive regulation of calcium ion-dependent exocytosis, regulation of cardiac muscle contraction by regulation of the release of sequestered calcium ion, calcium-dependent cell-cell adhesion via plasma membrane cell adhesion molecules for GO biological process (Figure 4C). Beta-adrenergic blockers and calcium channel blockers act as two kinds of the drugs for ATAAD. Beta-adrenergic blockers, which controlling both heart rate and blood pressure, improved survival in ATAAD patients overall and who received surgery (Suzuki et al., 2012) However, there is still a lack of high-quality, random trials to evaluate the long-term efficacy of beta adrenergic-blocker in AD treatment (Koo et al., 2017). Calcium channel blockers have been shown to selectively improve survival only in type B AAD patients (Suzuki et al., 2012). We conjectured that calcium related signaling might be closely related to the causes of ATAAD. Our results suggested potential roles of beta-blocker and calcium channel blockers in the prevention and treatment of ATAAD.

Surgery is the most important treatment for ATAAD. Due to the high mortality, drug treatment before the onset and after surgery should be significant for reducing mortality and recurrence of ATAAD. Here we predicted the potential drugs based on integrated analysis of consensus DEGs. The predicted compounds mainly included VEGFR inhibitor, HDAC inhibitors, HSP inhibitor, calcium channel blocker and glucocorticoid receptor agonist (Figure 5B). As mentioned above, calcium channel blocker TL_HRAS26 which regulates calcium signals might act as a potential drug for ATAAD. A recent study showed that glucocorticoids regulate the vascular remodeling of aortic dissection, (Zhang et al., 2018) our results also indicated that hydrocortisone hemisuccinate might be a useful drug by activating glucocorticoid receptor. Previous research demonstrated that aorta samples are mainly made up of aortic smooth muscle cells, fibroblasts and endothelial cells. We speculated that VEGFR inhibitor might regulate the endothelial cells in the aorta in ATAAD treatment(Pan et al., 2017). Although there is no evidence for using the remaining compound categories in ATAAD, our results provided new insights and possibilities for drug treatment.

miRNAs alternation in serum and aortic tissues have been reported to contribute to the diagnosis and development of acute aortic dissection (Liao et al., 2011; Wang et al., 2015; Dong et al., 2017; Wang T et al., 2017; Xu et al., 2017; Sbarouni and Georgiadou, 2018b). Here, we identified seven upregulated differentially expressed miRNAs including miR-215, miR-181c-3p, miR-7-1-3p, miR-374c-5p, miR-214-5p, miR-193a-3p, miR-365a-3p from GSE98770 (Figure 6A). The roles of these miRNAs are still unclear in ATAAD. Integrative analysis revealed the interaction of miRNA and targeted DEG. Altogether 16 targeted DEGs were filtered. Consistent with previous studies, (Kimura et al., 2017; Corbitt et al., 2018) our data implied that TIMP3 might take an important role in ATAAD pathogenesis. The downregulated DEG *JAK2* in our study has been demonstrated to have complex effects on aortic disease. Previous study of GSE52093 hypothesized that chronic inflammation in the aortic wall leading by decreased JAK2 expression contributed to ATAAD (Pan et al., 2014). However, JAK2 activation by increasing JAK2 phosphorylation was found in angiotensin II treated murine vascular smooth muscle cells (VSMCs), namely the *in vitro* model of dissecting aneurysm recently (Tanaka et al., 2018). Therefore, the functional role of JAK2 in ATAAD still needs further exploration. The roles of remaining targeted DEGs in ATAAD are still unclear, and these genes might be novel and potential targets of ATAAD.

At last, we validated 5 master DEGs in the human ATAAD sample and AD cell model. *SORBS1* and *TIMP3* were downregulated in ATAAD samples and AD models *in vitro*, while *PRUNE2* only decreased in AD cell model (Figure 7). Among the predicted compounds, TL_HRAS26 modulated all three master DEGs, hydrocortisone hemisuccinate regulated *SORBS1* and *TIMP3* at the same times. These results implied that calcium channel blocker TL_HRAS26 and hydrocortisone hemisuccinate might be potential drugs for prevention of ATAAD and recurrence after surgery. On the other hand, we did not find a difference in *PRUNE2*, *ALDH2* and *DMD in vivo* ATAAD samples and *in vitro* AD model, while *SORBS1* downregulated only in AD cell model. We hypothesized that this might be due to the individual differences or a small sample size of *in vivo* ATAAD samples.

## Conclusion

In conclusion, the present study offers potential targets and underlying molecular mechanisms ATAAD pathogenesis, prevention and drug discovery.

## Data Availability Statement

The original contributions presented in the study are included in the article/Supplementary Material, further inquiries can be directed to the corresponding author.

## Ethics Statement

The study was conducted under prior informed consent procedure and with written consent from the human ethics committee of the first affiliated hospital, college of medicine, Zhejiang University, China. (No.2019-1493)

## Author Contributions

All authors have made a substantial, direct, and intellectual contribution to the work and approved it for publication.

## Conflict of Interest

The authors declare that the research was conducted in the absence of any commercial or financial relationships that could be construed as a potential conflict of interest.

## References

[B1] Almontashiri N. A. Antoine D. Zhou X. Vilmundarson R. O. Zhang S. X. Hao K. N. (2015). 9p21.3 coronary artery disease risk variants disrupt tead transcription factor-dependent transforming growth factor beta regulation of p16 expression in human aortic smooth muscle cells. Circulation. 132, 1969–1978. 10.1161/CIRCULATIONAHA.114.015023 26487755

[B2] Ashburn T. T. Thor K. B. (2004). Drug repositioning: identifying and developing new uses for existing drugs. Nat. Rev. Drug Discov. 3, 673–683. 10.1038/nrd1468 15286734

[B3] Bossone E. LaBounty T. M. Eagle K. A. (2018). Acute aortic syndromes: diagnosis and management, an update. Eur. Heart J. 39, 739–749. 10.1093/eurheartj/ehx319 29106452

[B4] Cheuk B. L. Cheng S. W. (2011). Differential expression of elastin assembly genes in patients with stanford type a aortic dissection using microarray analysis. J. Vasc. Surg. 53, 1071–1078. 10.1016/j.jvs.2010.11.035 21276682

[B5] Corbitt H. Morris S. A. Gravholt C. H. Mortensen K. H. Tippner-Hedges R. Silberbach M. (2018). Timp3 and timp1 are risk genes for bicuspid aortic valve and aortopathy in turner syndrome. PLoS Genet. 14, e1007692 10.1371/journal.pgen.1007692 30281655 PMC6188895

[B6] Dong J. Bao J. Feng R. Zhao Z. Lu Q. Wang G. (2017). Circulating micrornas: a novel potential biomarker for diagnosing acute aortic dissection. Sci. Rep. 7, 12784 10.1038/s41598-017-13104-w 28986538 PMC5630636

[B7] Duan Q. Reid S. P. Clark N. R. Wang Z. Fernandez N. F. Rouillard A. D. (2016). L1000cds^2^: lincs l1000 characteristic direction signatures search engine. Npj Syst. Biol. Appl. 2 10.1038/npjsba.2016.15 PMC5389891 28413689

[B8] Erbel R. Aboyans V. Boileau C. Bossone E. Bartolomeo R. D. Eggebrecht H. (2014). 2014 esc guidelines on the diagnosis and treatment of aortic diseases: document covering acute and chronic aortic diseases of the thoracic and abdominal aorta of the adult. The task force for the diagnosis and treatment of aortic diseases of the european society of cardiology (esc). Eur. Heart J. 35, 2873–2926. 10.1093/eurheartj/ehu281 25173340

[B9] Evangelista A. Isselbacher E. M. Bossone E. Gleason T. G. Eusanio M. D. Sechtem U. (2018). Insights from the international registry of acute aortic dissection: a 20-year experience of collaborative clinical research. Circulation. 137, 1846–1860. 10.1161/CIRCULATIONAHA.117.031264 29685932

[B10] Golledge J. Eagle K. A. (2008). Acute aortic dissection. Lancet. 372, 55–66. 10.1016/S0140-6736(08)60994-0 18603160

[B11] Hagan P. G. Nienaber C. A. Isselbacher E. M. Bruckman D. Karavite D. J. Russman P. L. (2000). The international registry of acute aortic dissection (irad): new insights into an old disease. Jama. 283, 897–903. 10.1001/jama.283.7.897 10685714

[B12] Horita H. Wysoczynski C. L. Walker L. A. Moulton K. S. Li M. Ostriker A. (2016). Nuclear pten functions as an essential regulator of srf-dependent transcription to control smooth muscle differentiation. Nat. Commun. 7, 10830 10.1038/ncomms10830 26940659 PMC5411712

[B13] Huang J. Wang T. Wright A. C. Yang J. Zhou S. Li L. (2015). Myocardin is required for maintenance of vascular and visceral smooth muscle homeostasis during postnatal development. Proc. Natl. Acad. Sci. U. S. A. 112, 4447–4452. 10.1073/pnas.1420363112 25805819 PMC4394251

[B14] Karaman B. Sippl W. (2018). Computational drug repurposing: current trends. Curr. Med. Chem. 26, 5389–5409. 10.2174/0929867325666180530100332 29848268

[B15] Kimura N. Futamura K. Arakawa M. Okada N. Emrich F. Okamura H. (2017). Gene expression profiling of acute type a aortic dissection combined with *in vitro* assessment. Eur. J. Cardio. Thorac. Surg. 52, 810–817. 10.1093/ejcts/ezx095 28402522

[B16] Kiris I. Kapan S. Kilbas A. Yilmaz N. Altuntaş I. Karahan N. (2008). The protective effect of erythropoietin on renal injury induced by abdominal aortic-ischemia-reperfusion in rats. J. Surg. Res. 149, 206–213. 10.1016/j.jss.2007.12.752 18639893

[B17] Koo H. K. Lawrence K. A. Musini V. M. (2017). Beta-blockers for preventing aortic dissection in marfan syndrome. Cochrane Database Syst. Rev. 11, D11103 10.1002/14651858.CD011103.pub2 PMC6486285 29110304

[B18] Li B. Wang Z. Hu Z. Zhang M. Ren Z. Zhou Z. (2017). P38 mapk signaling pathway mediates angiotensin ii-induced mir143/145 gene cluster downregulation during aortic dissection formation. Ann. Vasc. Surg. 40, 262–273. 10.1016/j.avsg.2016.09.016 28167124

[B19] Li L. Pan Z. Yang X. (2018). Key genes and co-expression network analysis in liver of type 2 diabetes. J. Diabetes Investig. 10, 951–962. 10.1111/jdi.12998 PMC6626963 30592156

[B20] Liao M. Zou S. Weng J. Hou L. Yang L. Zhao Z. (2011). A microrna profile comparison between thoracic aortic dissection and normal thoracic aorta indicates the potential role of micrornas in contributing to thoracic aortic dissection pathogenesis. J. Vasc. Surg. 53, 1341–1349. 10.1016/j.jvs.2010.11.113 21334170

[B21] Mussa F. F. Horton J. D. Moridzadeh R. Nicholson J. Trimarchi S. Eagle K. A. (2016). Acute aortic dissection and intramural hematoma: a systematic review. Jama. 316, 754–763. 10.1001/jama.2016.10026 27533160

[B22] Nienaber C. A. Clough R. E. (2015). Management of acute aortic dissection. Lancet. 385, 800–811. 10.1016/S0140-6736(14)61005-9 25662791

[B23] Olsson C. Thelin S. Stahle E. Ekbom A. Granath F. (2006). Thoracic aortic aneurysm and dissection: increasing prevalence and improved outcomes reported in a nationwide population-based study of more than 14,000 cases from 1987 to 2002. Circulation. 114, 2611–2618. 10.1161/CIRCULATIONAHA.106.630400 17145990

[B24] Pan S. Lai H. Shen Y. Breeze C. Beck S. Hong T. (2017). Dna methylome analysis reveals distinct epigenetic patterns of ascending aortic dissection and bicuspid aortic valve. Cardiovasc. Res. 113, 692–704. 10.1093/cvr/cvx050 28444195

[B25] Pan S. Wu D. Teschendorff A. E. Hong T. Wang L. Qian M. (2014). Jak2-centered interactome hotspot identified by an integrative network algorithm in acute stanford type a aortic dissection. PloS One. 9, e89406 10.1371/journal.pone.0089406 24586754 PMC3933461

[B26] Pape L. A. Awais M. Woznicki E. M. Suzuki T. Trimarchi S. Evangelista A. (2015). Presentation, diagnosis, and outcomes of acute aortic dissection: 17-year trends from the international registry of acute aortic dissection. J. Am. Coll. Cardiol. 66, 350–358. 10.1016/j.jacc.2015.05.029 26205591

[B27] Sbarouni E. Georgiadou P. (2018a). Micrornas in acute aortic dissection. J. Thorac. Dis. 10, 1256–1257. 10.21037/jtd.2018.03.27 29708184 PMC5906359

[B28] Sbarouni E. Georgiadou P. (2018b). Micrornas in acute aortic dissection. J. Thorac. Dis. 10, 1256–1257. 10.21037/jtd.2018.03.27 29708184 PMC5906359

[B29] Suzuki T. Isselbacher E. M. Nienaber C. A. Pyeritz R. E. Eagle K. A. Tsai T. T. (2012). Type-selective benefits of medications in treatment of acute aortic dissection (from the international registry of acute aortic dissection [irad]). Am. J. Cardiol. 109, 122–127. 10.1016/j.amjcard.2011.08.012 21944678

[B30] Tanaka T. Kelly M. Takei Y. Yamanouchi D. (2018). Rankl-mediated osteoclastogenic differentiation of macrophages in the abdominal aorta of angiotensin ii-infused apolipoprotein e knockout mice. J. Vasc. Surg. 68, 48–59. 10.1016/j.jvs.2017.11.091 PMC6558660 29685509

[B31] Wang L. Zhang S. Xu Z. Zhang J. Li L. Zhao G. (2017). The diagnostic value of microrna-4787-5p and microrna-4306 in patients with acute aortic dissection. Am. J. Transl. Res. 9, 5138–5149. 29218111 PMC5714797

[B32] Wang T. He X. Liu X. Liu Y. Zhang W. Huang Q. (2017). Weighted gene co-expression network analysis identifies fkbp11 as a key regulator in acute aortic dissection through a nf-kb dependent pathway. Front. Physiol. 8, 1010 10.3389/fphys.2017.01010 29255427 PMC5723018

[B33] Wang X. J. Huang B. Yang Y. M. Zhang L. Su W. J. Tian L. (2015). Differential expression of micrornas in aortic tissue and plasma in patients with acute aortic dissection. J Geriatr Cardiol. 12, 655–661. 10.11909/j.issn.1671-5411.2015.06.013 26788043 PMC4712372

[B34] Weis-Muller B. T. Modlich O. Drobinskaya I. Unay D. Huber R. Bojar H. (2006). Gene expression in acute stanford type a dissection: a comparative microarray study. J. Transl. Med. 4, 29 10.1186/1479-5876-4-29 16824202 PMC1557406

[B35] Xu H. Du S. Fang B. Li C. Jia X. Zheng S. (2019). Vsmc-specific ep4 deletion exacerbates angiotensin ii-induced aortic dissection by increasing vascular inflammation and blood pressure. Proc. Natl. Acad. Sci. U. S. A. 116, 8457–8462. 10.1073/pnas.1902119116 30948641 PMC6486760

[B36] Xu Z. Wang Q. Pan J. Sheng X. Hou D. Chong H. (2017). Characterization of serum mirnas as molecular biomarkers for acute stanford type a aortic dissection diagnosis. Sci. Rep. 7, 13659 10.1038/s41598-017-13696-3 29057982 PMC5651857

[B37] Zhang L. Zhou J. Jing Z. Xiao Y. Sun Y. Wu Y. (2018). Glucocorticoids regulate the vascular remodeling of aortic dissection via the p38 mapk-hsp27 pathway mediated by soluble tnf-rii. Ebiomedicine. 27, 247–257. 10.1016/j.ebiom.2017.12.002 29287621 PMC5828293

[B38] Zhong X. X. Wei X. Jiang D. S. Zhu X. H. Liu L. G. (2020). [expression of tropomyosin 2 in aortic dissection tissue]. Zhonghua Xinxueguanbing Zazhi. 48, 777–781. 10.3760/cma.j.cn112148-20200707-00540 32957762

