# Supplementary material for: Regulatory Master Genes Identification and Drug Repositioning by Integrative mRNA-miRNA Network Analysis for Acute Type A Aortic Dissection
Source: Front Pharmacol. 2021 Jan 21;11:575765. doi: 10.3389/fphar.2020.575765 (PMC7861055; doi:10.3389/fphar.2020.575765)
Supplement: Supplementary file 1 [file table1.docx]

Supplemental table 1. Primers and conditions for qRT-PCR

| Genes | Primers (5’-3’) |
| --- | --- |
| *PRUNE2* | CTGGGTCTCCAGAGGATGAA  TGGTTTCCCTCACTTCTTCG |
| *ALDH2* | TCATCATGTCAGATGCCGATA  TCCTCCTGCACGAAGGTC |
| *SORBS1* | TCCTGAAATCCAGCAAACTTC  TGCAGTGAGCTATGACTGTACCA |
| *DMD* | TCAGACAATTCAGCCCAGTCT  CAAACTCTGGCTCTGCTTCA |
| *TIMP3* | GAGCTGCCAATTGAAACAGA  GCACTTGTGTGTGTGCAAGA |
| *GAPDH* | GCACCGTCAAGGCTGAGAAC  TGGTGAAGACGCCAGTGGA |
